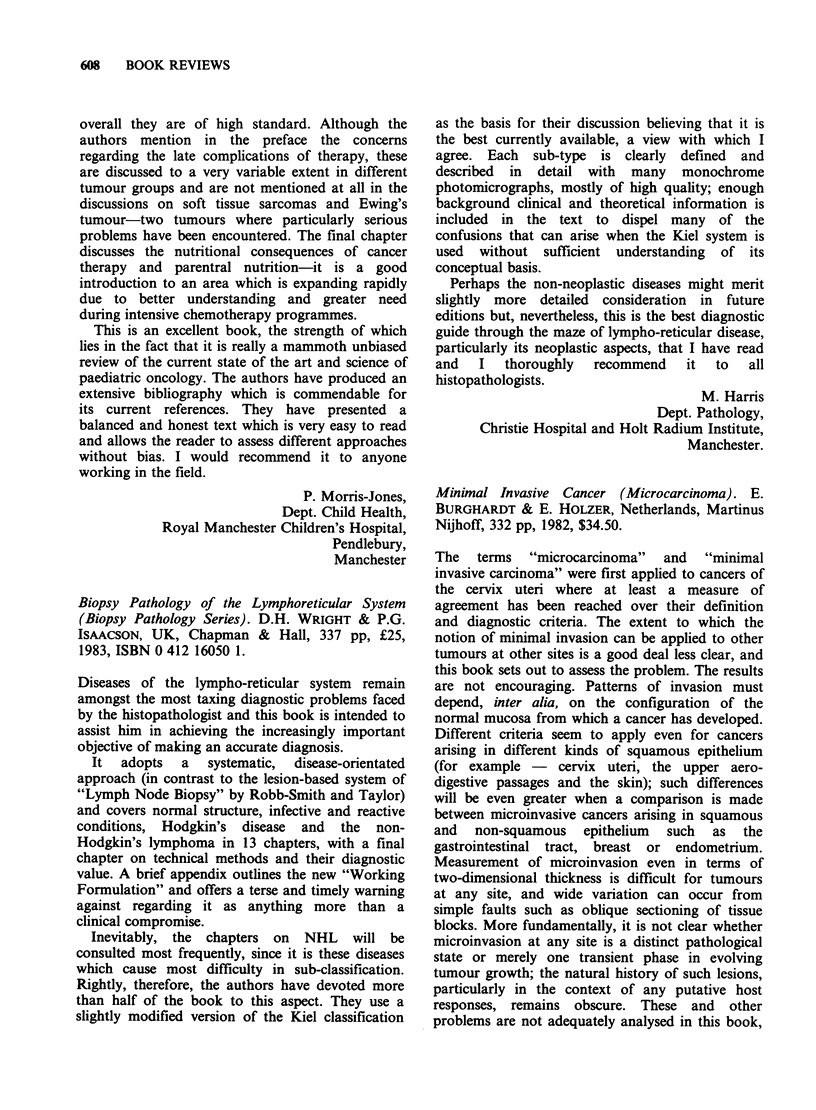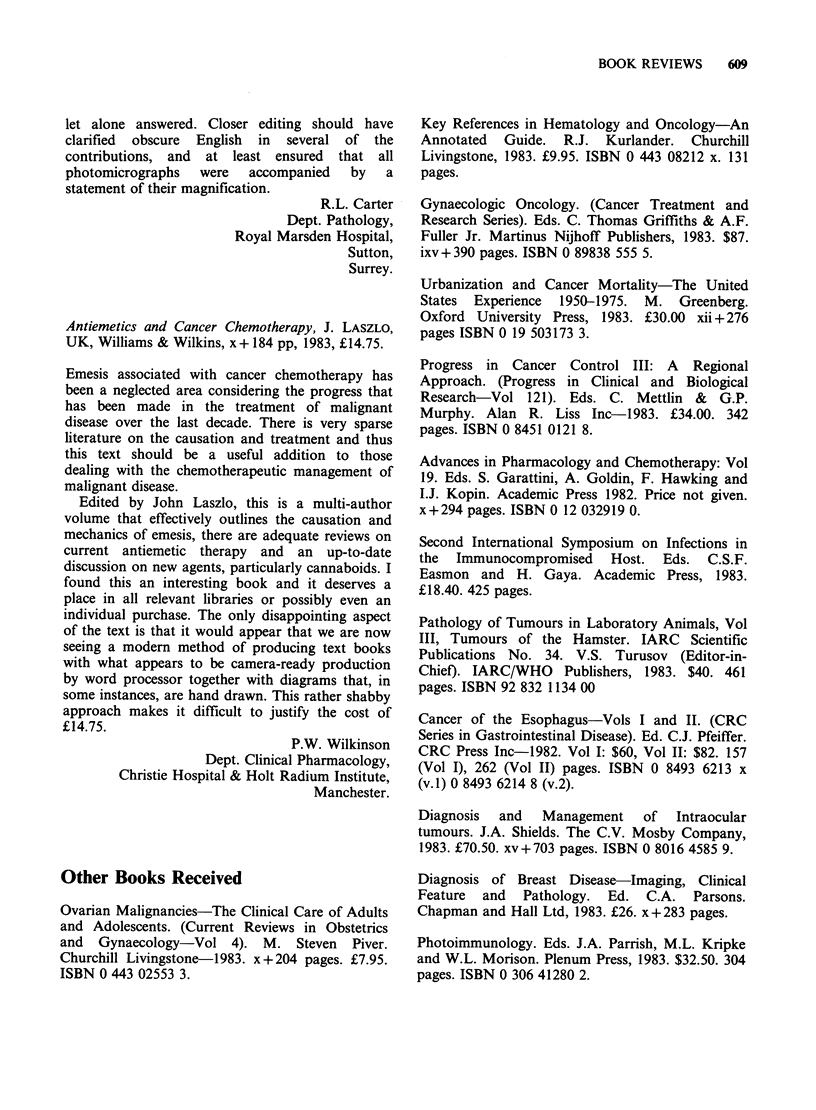# Minimal Invasive Cancer (Microcarcinoma)

**Published:** 1983-10

**Authors:** R.L. Carter


*Minimal Invasive Cancer (Microcarcinoma).* E. BURGHARDT & E. HOLZER, Netherlands, Martinus Nijhoff, 332 pp, 1982, $34.50.

The terms "microcarcinoma" and "minimal invasive carcinoma" were first applied to cancers of the cervix uteri where at least a measure of agreement has been reached over their definition and diagnostic criteria. The extent to which the notion of minimal invasion can be applied to other tumours at other sites is a good deal less clear, and this book sets out to assess the problem. The results are not encouraging. Patterns of invasion must depend, *inter alia,* on the configuration of the normal mucosa from which a cancer has developed. Different criteria seem to apply even for cancers arising in different kinds of squamous epithelium (for example — cervix uteri, the upper aero-digestive passages and the skin); such differences will be even greater when a comparison is made between microinvasive cancers arising in squamous and non-squamous epithelium such as the gastrointestinal tract, breast or endometrium. Measurement of microinvasion even in terms of two-dimensional thickness is difficult for tumours at any site, and wide variation can occur from simple faults such as oblique sectioning of tissue blocks. More fundamentally, it is not clear whether microinvasion at any site is a distinct pathological state or merely one transient phase in evolving tumour growth; the natural history of such lesions, particularly in the context of any putative host responses, remains obscure. These and other problems are not adequately analysed in this book, let alone answered. Closer editing should have clarified obscure English in several of the contributions, and at least ensured that all photomicrographs were accompanied by a statement of their magnification.

R.L. Carter
Dept. Pathology,
Royal Marsden Hospital,
Sutton,
Surrey.